# Flow-induced platelet activation in components of the extracorporeal membrane oxygenation circuit

**DOI:** 10.1038/s41598-018-32247-y

**Published:** 2018-09-18

**Authors:** Gabriel Fuchs, Niclas Berg, L. Mikael Broman, Lisa Prahl Wittberg

**Affiliations:** 1 Department of Physiology and Pharmacology, Karolinska Institutet, Stockholm, Sweden; 2 Department of Cardiology, Sundsvall Regional Hospital, Sundsvall, Sweden; 3 Linné Flow Centre & BioMEx, KTH Mechanics, Royal Institute of Technology (KTH), Stockholm, Sweden; 4 ECMO Centre Karolinska, Pediatric Perioperative Medicine and Intensive Care, Karolinska University Hospital, Stockholm, Sweden

## Abstract

Extracorporeal membrane oxygenation (ECMO) is used for rescue in severe respiratory and/or circulatory failure. The patient’s blood is pumped over artificial surfaces in the ECMO circuit. A platelet activation model was applied to study the potential thrombogenicity of ECMO circuit components: the centrifugal blood pump, cannulae, and tubing connectors. Based on the accumulated effect of the scalar form of the stress acting on the platelet over time, the activation model enables assessment of platelet activation and pinpoints regions of elevated activation risk in a component. Numerical simulations of the flow in different components of the ECMO circuit was carried out where the activation level is a function of the impact of local stress and its history along the path that the platelets follow. The results showed that the pump carried the largest risk for platelet activation followed by the reinfusion cannula and lastly the connectors. Pump thrombogenicity was mainly due to long residence time and high shear-rate while the connector showed a high level of non-stationary shear-rate that in turn may contribute to the formation of aggregates through direct platelet activation or through high shear-rate modulation of the vWF multimers.

## Introduction

Extracorporeal Membrane Oxygenation (ECMO) is a life-saving therapy for temporary support in refractory severe lung and/or heart failure. ECMO treatment has in recent years seen increased use and improved results in the intensive care setting. Moreover, ECMO devices are becoming more widely used also outside intensive care units as extracorporeal cardiopulmonary resuscitation for refractory cardiac arrest^1^. However, the success of ECMO is associated with the use of anticoagulation by heparin or direct thrombin inhibitors in a balance between thromboembolic events and increased risk for bleedings.

The ECMO circuit is composed of a centrifugal pump, a membrane oxygenator (artificial lung), cannulas implanted into the patient’s major vessels, and tubing along with multiple tubing connectors. Thus, the blood is exposed to large area of artificial surfaces under non-physiological conditions promoting coagulation activation. Although anti-coagulation is routinely used intravenously (i.v.) and in the lining materials for biocompatibility, thromboembolic events may still occur. Together with bleeding, thromboembolic events constitute the main complications associated with ECMO treatment. The approach of i.v. anticoagulation has been in use over decades in different situations, such as during coronary artery bypass grafting^2^. Johnell *et al*.^2^ found that low-dose systemic heparin may not be sufficient to maintain adequate antithrombotic activity. More importantly, it was also observed that high-dose heparin resulted in direct cell activation rather than increased anti-coagulation and anti-inflammatory effect. In a retrospective study, Trudzinski *et al*.^3^ reported an incidence of 46.1% for venous thrombosis and/or thromboembolism in patients treated on ECMO with systemic anticoagulation.

It is commonly recognized that the main source of enhanced thrombogenicity in the ECMO circuit is of mechanical origin, initiating a vicious biochemical circle. The centrifugal pump used in ECMO has been the topic for a considerable number of both experimental *in-vitro* and animal studies with the aim to minimize the risk for thrombus formation. Mueller *et al*.^4^ reported clinical use of the CentriMag (Thoratec Corporation, Pleasanton, CA, USA) centrifugal pump during beating-heart coronary artery bypass grafting in 11 patients. Heparin was given to maintain adequate coagulation time. The number of thrombi was reported and a marginal hemolysis was observed from assessment of plasma free hemoglobin. Fujiwara *et al*.^5^ carried out animal experiments with magnetically levitated centrifugal blood pumps (MedTech Mag-Lev, Medtronic Bio-Pump BPX-80), revealing a major issue of severe hemolysis and thrombus formation in spite of a well-controlled anticoagulation by heparin. Chan *et al*.^6^ carried out experiments using the CentriMag to evaluate its biocompatibility. It was found that leukocytes and red blood cells (RBC) were damaged along with formation of micro-particles and cleavage of vWF (von Willebrand factor) multimers. Hastings *et al*.^7^ showed that the tube to connector junction was a very common site for finding thrombi. They also reported that the anchored thrombi at this location were fibrin and RBC rich.

Modeling platelet activation has been initiated in order to assess the relative performance and biocompatibility for extracorporeal as well as implanted blood pumps. Bluestein and co-workers published a sequence of papers reporting the development of a platelet activation model (Nobili *et al*.^8^, Soares *et al*.^9^). The model is of power-law type, based on a scalar form (magnitude) of the shear-stress tensor. Moreover, the model accounts for history effects by accumulating the stress along the platelet’s path and by including the time derivative of the stress. Numerical simulations were complimented by *in-vitro* platelet activation measurements. Bluestein *et al*.^10^ also developed a thrombogenicity emulation (DTE) methodology for optimizing device resistance to thrombus formation, demonstrating that optimizing devices with respect to exposure to levels of elevated shear stresses as well as exposure times may reduce shear-induced platelet activation. Furthermore, the same group introduced the *Platelet Activity State* (PAS) as a measure for platelet activation. PAS is a parameter normalized with respect to the maximum thrombin generation of fully-activated platelets. The approach has been applied for the assessment of two implantable blood pumps (ventricular assist devices) by Chiu *et al*.^11^.

In the current study, the model by Nobili *et al*.^7^ is applied in order to assess the level of platelet activation in different ECMO circuit components. The focus is not only on the distribution of platelets associated with a certain PAS, but also on the time platelets remain in the flow found in the different components and how this residence time is affected by different rotational pump speeds.

## Material and Methods

In this work, three components of the ECMO circuit are investigated; a centrifugal pump, ECMO cannulae used for drainage and reinfusion, and the straight tubing connector.

**Figure 1:**
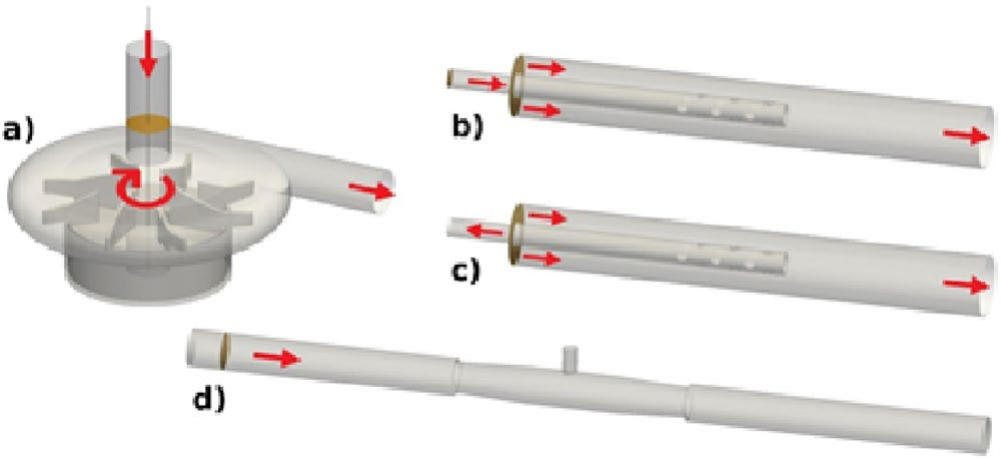
Geometry of the pump (**a**), reinfusion cannula (**b**), drainage cannula (**c**) and a tube connector (**d**). Arrows indicate flow or rotational direction, respectively. The platelet injection planes are depicted by orange surfaces. Note the lighthouse tip design of the cannula in (**b**) and (**c**).

The pump (Fig. 1a) is characterized by an impeller having 4 larger and 4 smaller blades, similar to geometry as studied by Zhang *et al*.^12^. The impeller diameter is approximately 44 mm. To the impeller, an annular magnet house having a central hole of 9 mm in diameter and a height of 12 mm is attached. The impeller is placed in the pump house leaving a gap of 0.5 mm in radial and 1 mm axial direction between the impeller magnet and the pump house, respectively. The impeller is levitating in a magnetic field. Thus, no contact to the pump housing occurs.

The cannula used was a Bio-Medicus^TM^ (Medtronic Europe Sárl, Tolochenaz, Switzerland) 23 French (Fr), with a 3/8” connector and *lighthouse tip*, Fig. 1b,c. In the simulations, the cannula was placed in a pipe with a radius of 10 mm in order to model the cannula in the Inferior vena cava (IVC). The cannula size given in Fr refers to the outer diameter and is converted to millimeters by dividing Fr by 3 (1 Fr = 1/3 mm). The length of the cannula was 25 inner diameters in order to allow the flow to develop within the cannula to avoid strong effect on the flow at the tip imposed by the inflow condition. Similarly, the outer pipe was extended so that the inflow and outflow conditions had minor effect on the flow around the tip of the cannula.

The 3/8″ straight connector (Fig. 1d) has conical inlet- and outlet segments with outer and inner diameters of 11 and 9 mm, respectively. The inlet and outlet sections of the connector and cannulae were extended 20 inner diameters to minimize the influence of inlet and outlet conditions on the turbulent flow in the connector and the proximal region surrounding it.

The respective shape of the pump, cannulae and the connector was used to generate the computational domain. The inlet and outlet of the pump and the connector have similar conical shapes allowing for easy and good connection to the 3/8″ PVC tubing. At the region of the connection between the pump or the connector and the tube, there is an expansion due to the conical shape and the finite thickness of the lip of the connector. This expansion is a critical point as it induces unsteady turbulent flow separation as will be shown further on. The size of the expansion is less than 1 mm.

The geometry of the pump was extended at the inlet (10 pipe diameters) and outlet (15 pipe diameters). These extensions are needed in order to set boundary conditions allowing for natural development of the flow. For the pump computations, a sliding grid approach was applied having one grid attached to, and rotating with, the impeller whereas a second grid was attached to the pump house remaining stationary. Interpolation was used at the interface between the two grids such that the rotation of the impeller was accounted for. The total number of computational grid points were eight, three and one Million for the pump, connector and the cannulae, respectively. Higher resolution was used at regions of high shear. The flow turned out to be turbulent in the pump and the connector while it showed transitional behavior in the cannulae. The governing equations (Navier-Stokes) were discretized by finite-volume approach with formally second order accuracy. The resulting set of algebraic equations were integrated in time using an implicit method. The time resolution was such that turbulent fluctuations were well resolved and hence no explicit turbulence model was used. Thus, the solutions correspond to Direct Numerical Simulation (DNS) when all the scales are resolved and to Large Eddy Simulations when a small portion of the turbulent spectrum remains unresolved. An estimate of the level of grid resolution can be obtained by considering the spectrum of the kinetic energy of the flow at different locations in the flow field. These spectra show that the resolution was such that a portion of the inertial subrange was resolved. Due to the fact that the flow was laminar, transitional and turbulent in different parts of the computational domain it is impossible to infer that the results are full DNS. The sensitivity of the results with respect to grid resolution was assessed by resolving the flow in the pump for three different girds: coarse, medium and fine grids, corresponding to four, eight and 16 Million computational cells, respectively. The deviation between the medium-fine grid results, in terms of the time or phase average velocity data normalized with the inlet velocity, was less than 2%. All the results presented in the following have been computed using the STAR-CCM+ CFD package.

In all simulations, the fluid was assumed to be incompressible with blood assumed to be Newtonian having density of 998 kg/m^3^ and dynamic viscosity of 8.89 10^−4^ Pa s. In all cases, the same inlet and outlet boundary conditions were used. At the inlet boundary, constant velocity was imposed on the axial component and zero on the other two velocity vector components. Additionally, a synthetic turbulence with 5% intensity and a length scale of 0.5 mm was added to the inlet velocity vector. The volume flow rate was kept constant in the results presented here (4 L/min). The length of the inlet pipe allowed the flow to develop and become unsteady. At the outlet boundary, a constant pressure was imposed. No-slip conditions were set at all solid surfaces that were both inlets or outlets.

For each of the configurations, a large number (100,000) particles were released into the mock circuit and followed while carried by the fluid. These particles, representing the platelets, followed to a large extent the instantaneous motion of the fluid due to their low Stokes number. The action of the stress on the platelet was expressed in terms of PAS. Statistical data, such as mean, minimal and peak values at the end of the simulation, for the platelet populations were computed in each respective ECMO component. Distribution functions of residence time and activation state was found by dividing the final PAS values into 25 equally spaced bins and counting the occurrence of values within each bin.

### Platelet activation model

The model used to assess the level of platelet activation is based on the approach of Nobili *et al*.^8^. PAS was defined as the number of activated platelets versus the total platelet population. The activation depends on the local instantaneous stress acting on the platelet as it moves in the fluid over time. The platelets are assumed to be small spherical and solid particles. The force responsible for the motion of the particle is assumed to be due to drag (equation 1). The platelets are small, having a Stokes number (defined as the ratio between the particle response time to changes in the flow to the local time scale of the flow) much less than unity (typically of the order of 10^−5^). This force leads to change in the platelet velocity and through equation (1) the position of the platelet can be found. The volume displaced by the platelets is small implying low platelet number density. Thus the total force that the particles exert on the fluid (and the same as the fluid exerts on the particle) is neglected (so called one-way interaction). The equation of motion is written:

1 $$\frac{d\,{{\bf{X}}}_{i}}{dt}={{\bf{U}}}_{i}\,\,{\rm{a}}{\rm{n}}{\rm{d}}\,\,{m}_{i}\,\frac{d\,{{\bf{U}}}_{i}}{dt}=6\pi \mu {R}_{i}[{\bf{u}}(t,{{\bf{X}}}_{i})-{{\bf{U}}}_{i}]$$

where ***m***_*i*_ is the mass, ***R***_*i*_ the radius, ***X***_*i*_ the position vector, and ***U***_*i*_ is the velocity vector of particle *i*. *µ* is the dynamic viscosity of the fluid and ***u*** is the fluid velocity at the particle location. The equation of motion was integrated by an explicit Euler method. The time-step was small enough both for stability reasons as well as to avoid the platelets to pass through the walls that may occur with too large time-steps. To circumvent this, a simple collision correction scheme was implemented. After each position update, an overlap test was performed between the points $[{X}_{i}^{n},{X}_{i}^{n+1}]$, where *X* with superscript *n* denotes the location of the platelet at time-step *n*. When these points happened to be on both sides of a solid wall (i.e. the particle crossed the wall) the time it takes to impact the wall was computed: ${\rm{\Delta }}t=\parallel {X}_{i}^{impact}-{X}_{i}^{n}\parallel \,/\,\parallel {U}_{i}^{n}\parallel$. This shorter time-step was used to re-compute the new platelet position. At impact, the platelet was assumed to bounce without losses.

The activation of platelets is assumed to obey a power law model, calibrated by empirical testing^8–11^. The model is based on the local stress (τ) acting on the platelet as it is transported by the fluid. Thus, PAS is evaluated by integrating in time together with the platelet motion

2 $$\frac{d\,\,{\rm{P}}{\rm{A}}{\rm{S}}}{dt}=c\,a\,{\tau }^{b/a}{D}^{a-1}\,\,{\rm{a}}{\rm{n}}{\rm{d}}\,\,\frac{d\,D}{dt}={\tau }^{b/a}$$

with *a, b* and *c* being model parameters, determined from empirical data (Bluestein *et al*.^10^), *τ* is the so called scalar stress (expressed in analogy to the Mises stress in solids). The numerical value of the model parameters (*a* = 1.3198, *b* = 0.6256, and *c* = 10^−5^), were not changed although one may argue that the flow conditions in the ECMO component differ from the original experimental conditions. Platelets are inserted into the inlet of each of the devices and are tracked while being carried by the blood flow. Depending on their initial location, the platelets stay within each of the devices over different times and are subject to local and time-dependent stress. Each of these tracked platelets can be followed and through equation (2), each particle’s residence time and level of PAS can be calculated.

After a transient period, the flow becomes statistically stationary for all cases. Platelet activation was analyzed only after reaching a statistically steady state. The computations were continued further and the instantaneous velocity and pressure fields were stored. The platelet activation model and the sensitivity analysis of model parameters was conducted on the stored data in a post-processing step.

## Results

The ECMO components under consideration are shown in Fig. 1, where the inlet and outflow planes are marked with arrows. The two cannula configurations correspond to blood co-flow for reinfusion (b), and counter-flow for the drainage (c) situation.

The rotation rate of the pump impeller was kept constant at 3,000 *rpm* with a flow rate of 4 L/min. The flow rate in the connector was also set to 4 L/min. For the two cannulae cases, the flow rates were 2 L/min and 4 L/min for the cannulae and outer pipe, respectively. In all simulations, the inlet tube was extended to enable the use of a top-hat profile at the entrance while allowing for the development of the flow in the extended sections of the inlet.

### Flow fields

**Figure 2:**
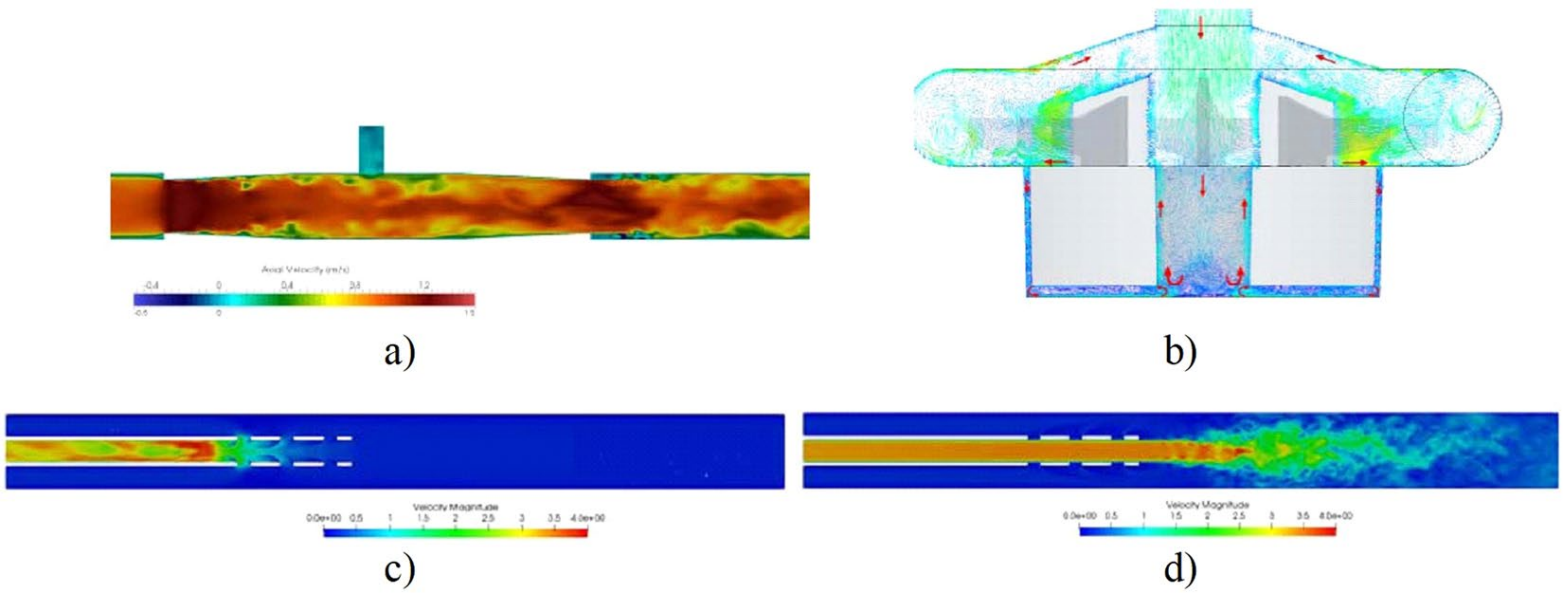
Instantaneous flow fields in the (**a**) the connector, (**b**) pump, (**c**) the drainage cannula and (**d**) the reinfusion cannula. Streamwise velocity levels are given by the color bars.

The flow in all configurations was unsteady leading to non-stationary, fluctuating stress in space and time. A typical flow field in the pump is displayed in Fig. 2. The rotating impeller produce a high-speed flow around the blades with a shear-layer at the edge and the tip of the blades. In the gap between the roof of the pump house and the blades, the flow is directed radially outwards closer to the blades and radially inwards near the roof of the pump house. The gap size between the blades and the roof was less than 4.5 mm. The peak shear-rate due to the blade rotation was found to reach values above 50,000 s^−1^.

The residence time within this region was estimated by the time that the platelets stayed in this region averaged over the platelet population. This residence time was greater than 0.2 s, i.e. more than 8 pump revolutions. The inlet fluid is mainly pulled into the flow generated by the blades, although part of the inlet fluid continues into the central hole of the magnet house. In this hole, the flow is deflected downwards centrally whereas displaying an upward motion near the rotating wall of the magnet. The axial flow was relatively slow in this region. However, due to the two abovementioned streams, the noted peak shear-rate reached values as high as 20,000 s^−1^. In the axially oriented, narrow gap between the magnet and the pump house, a set of about 5–6 azimuthally oriented wave vortices (alike the Taylor-Couette wavy vortices) was observed. A mean axial downwards directed flow was observed in the gap, having a mean flow rate of about 6 mL/s, corresponding to an averaged vertical velocity around 0.015 m/s. This slow vertical velocity implies that the mean residence time of the fluid trapped in this gap is about 0.1 s, equivalent to approximately four full impeller revolutions. The narrow gap between the rotating magnet house and the outer wall of the pump house yields high shear-rates with a peak of about 100,000 s^−1^. The narrow space found between the bottom part of the rotating magnet house and the pump house generates another set of vortices and slowly moving fluid. As the gap is narrow (of the order of 1 mm), strong shear-stress was expected, found to have a peak greater than 30,000 s^−1^ due to the rotation of the magnet house and the induced flow field. The flow near the rotating surface was directed radially outwards (centrifugal effect) while directed inwards near the stationary wall, as marked in Fig. 2a. This intra-gap circulation leads to relatively long residence times (of the order of 0.1 s) for the fluid in this region. At the outlet of the pump, where the outlet circular tube merges with the volute (the “tongue” of the pump) an unsteady flow separation was observed (not shown). This separated flow implied elevated shear-rate with peaks as large as 41,000 s^−1^. However, no real prolongation of the residence time was found as the separation bubble was washed away by the impeller blade passing, although reconstituted after the blade had passed.

The flow in the connector separates downstream of the connector lip. The gap between the connector lip and the tube wall was about 1 mm, where the shear-layer formed leads to strong spatial and temporal fluctuations with enhancement of the corresponding stress. The separation bubble was anchored to the connector lip but ended at a non-fixed downstream located stagnation point. Platelets that are normally found in higher concentration near the wall of the vessel were prone to be captured in the unsteady separation bubbles over a longer time as compared to platelets in the center of the connector. More importantly, the high amplitude fluctuations at the downstream side of the connector lead to strong shear-stress. Moreover, an almost completely stagnant region in the T-shaped *Luer-lock* injection port of the connector was noted, in turn leading to longer residence time for platelets captured within this region. The current results are supported by the clinical experience where fibrin formation/clots are often first detected at the downstream junction between the connector and the tubing. Clots are also frequently formed at the Luer-lock ports.

The cannula considered in this work has twelve small holes in addition to the hole at the end of the lumen. The flow through these holes must turn at least 90 degrees relative to the axial flow. This change of direction implies high level of shear and generation of small scale fluctuations. Hence, it is not of surprise noting that blood clots are commonly observed attached to the holes near the tip of the cannula. Furthermore, the character of the flow and the strength of the shear depend on the configuration of the cannula (i.e. drainage or reinfusion, respectively). The reinfusion case exhibits stronger shear as the jet emerging from the cannula into the lumen of the vessel interacts with a relatively strong co-axial uni-directional flow. The perpendicular jets emerging from the side holes thereby bend with the stream, producing a set of horse-shoe and wake vortices, alike those seen in a jet in cross flow. These vortices generated small scale fluctuations implying loss of energy as well as stronger shear.

### Platelet activation

The accumulated statistics of the platelets in each component is shown in Table 1. The simulation times corresponding to each case was long enough to generate reliable statistical data. The peak of PAS for the connector and cannulas were found similar. For the pump, PAS was significantly higher. The highest activation state, both in terms of maximum and mean value, was found in the pump. The tube connector and the drainage cannula showed similar activation levels, and below those of the reinfusion cannula. Note that the variation in PAS was large and the range differed by about four orders of magnitudes. This large variation depends on the history of each platelet, namely, the history of the level of the shear-stress along with its duration the platelet is subjected to. In the following, each of these components are characterized to better understand these results and relate them to the flow fields observed in the different cases.

**Table 1:** Summary of the platelet activation state (PAS) in the different cases.

Component	Simulation time (s)	PAS: Mean (range)
Pump (3,000 *rpm*)	0.8 s	1.3 10^−4^ (2.3 10^−7^ – 3.4 10^−2^)
Connector	10 s	8.8 10^−6^ (1.2 10^–7^ – 1.8 10^−3^)
Cannula (drainage)	17 s	8.4 10^−6^ (1.9 10^−7^ – 6.4 10^−3^)
Cannula (reinfusion)	20 s	9.6 10^−6^ (2.4 10^−7^ – 3.7 10^−3^)

The PAS values are given in terms of mean, minimal and maximal values.

**Figure 3:**
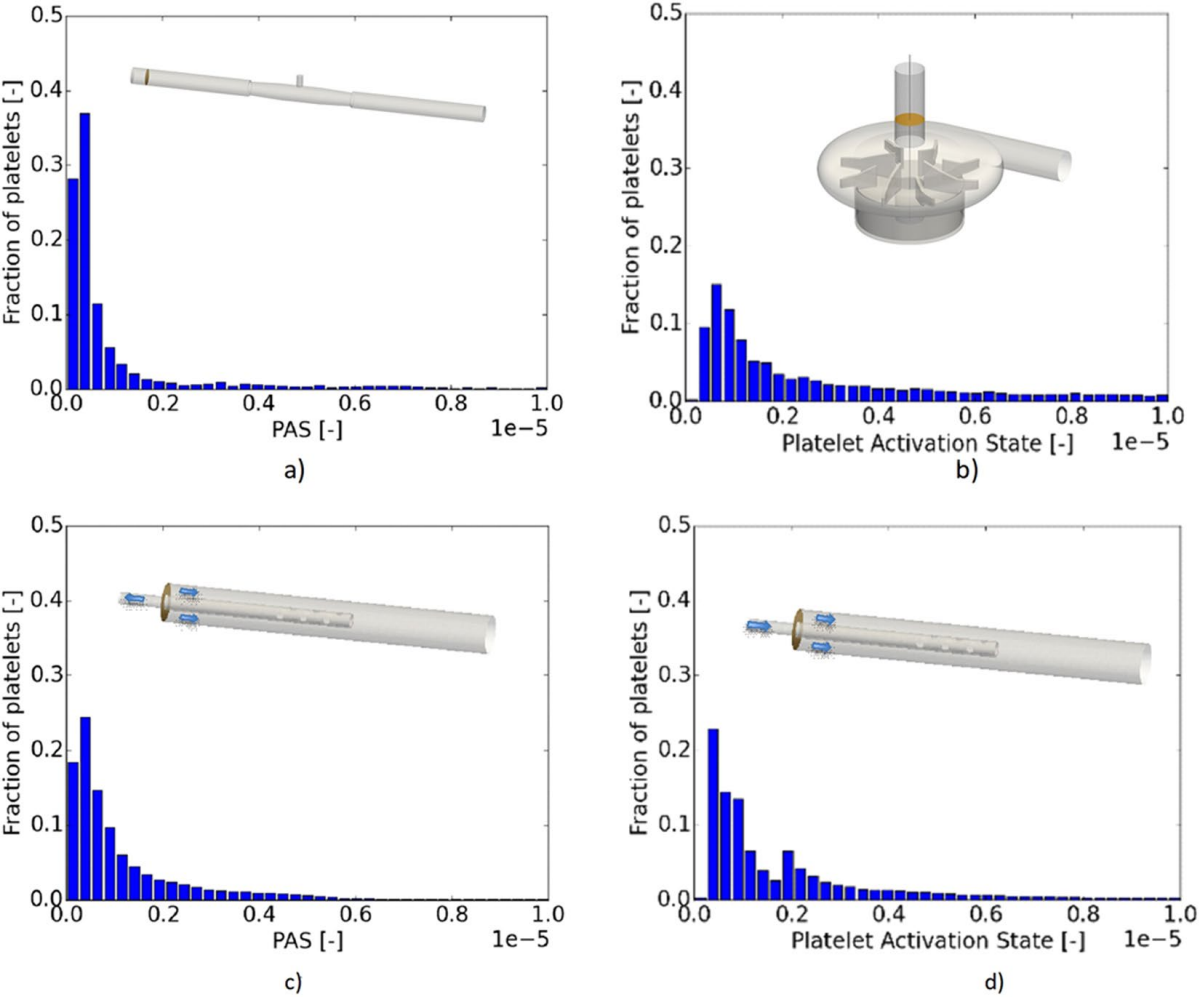
The fraction of the injected platelets vs level of activation (PAS) for four flow conditions: (**a**) the connector (**b**) the pump at 3,000 rpm, (**c**) the cannula draining the blood from the patient to the ECMO circuit and (**d**) the corresponding reinfusion case.

As noted, both the pump and the cannulae have a longer “tail” of activated platelets, expressing the fact that there is a higher fraction of more activated platelets as compared to the connector (Fig. 3). Similar to what has been reported in literature, the pump is a major risk for platelet activation. However, the other devices do contribute to the platelet activation. For the pump, the largest levels of PAS are found in the gap between the magnet and pump house as well as in the gap between the impeller and the roof of the pump house. In the space between the magnet and the pump house, undulating-wavy azimuthal vortices were formed as mentioned above (Taylor-Couette). Platelets captured in these vortices experienced both long residence times and strong shear-stress. Moreover, for mechanical pump efficiency the gap between the blades and the pump house is kept small, in turn leading to strong shear. The separated flow regions, some of which are shown in Fig. 2, contribute to both longer residence time and shear-stress fluctuations.

**Figure 4:**
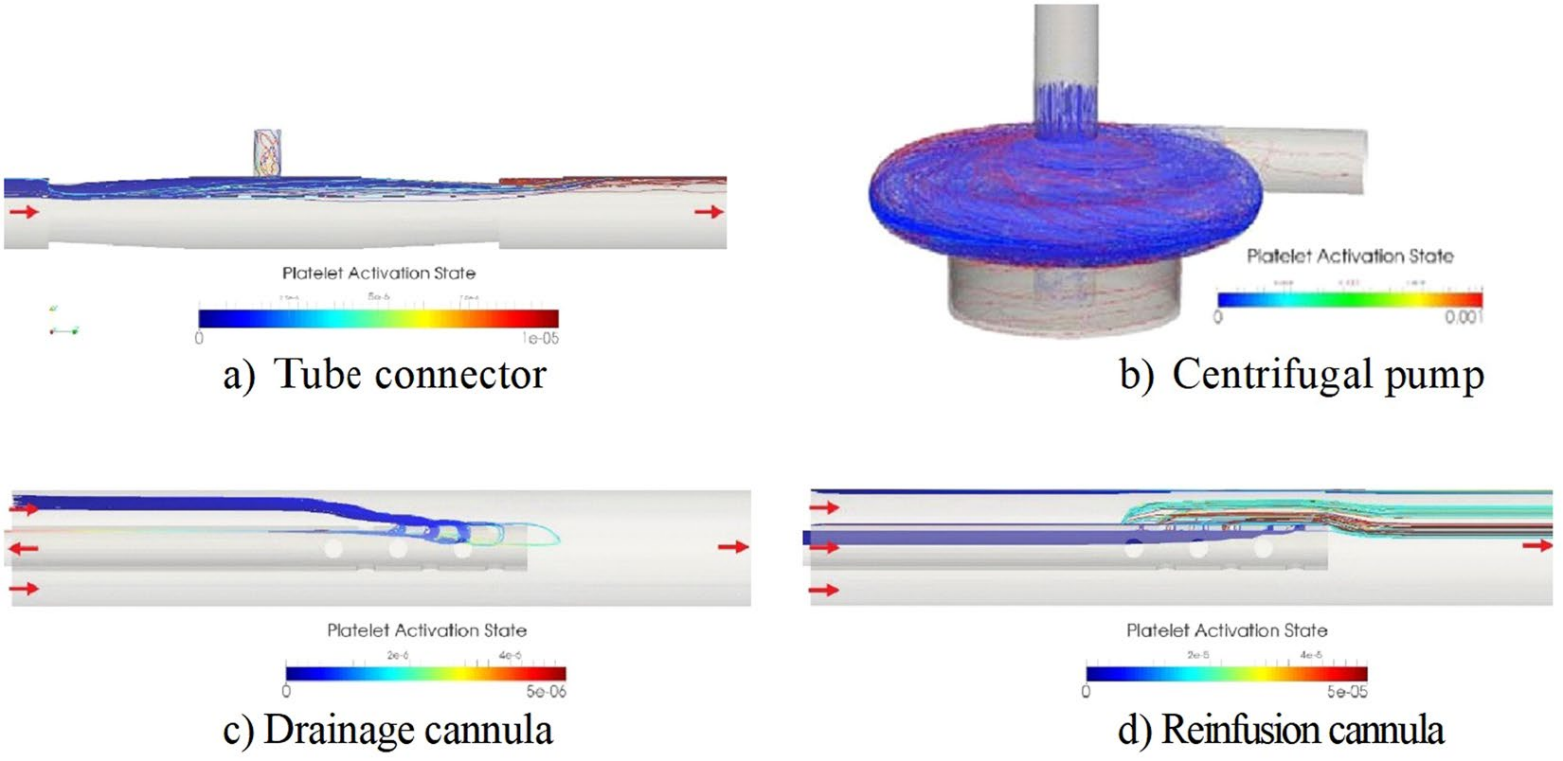
Platelets path-lines with the largest PAS values in the four cases. Red color implies large PAS value.

The connector showed a separated flow region downstream of the end of the conical connector and a stagnant region at the injection Luer-lock side tube (Fig. 4a). The downstream separation bubble was non-stationary that may lead to longer residence time for platelets near the wall of the tube. Moreover, the unsteady flow through the connector implies that platelets are captured and washed out after some time from the side tube. As noted, this effect leads to higher PAS value in the vicinity of the separation bubble found at the downstream part of the connector.

It can be noted that for the reinfusion cannula configuration (Fig. 4d), the flow through the side holes forces the fluid particles (“the platelets”) to undergo two strong bends terminating in the boundary layer around the cannula. This leads to strong shear stress and hence larger PAS values. In contrast, for the drainage cannula, the volume of the flow exiting via the end hole of the cannula was significantly greater as compared to the flow through the small side holes, implying lower PAS values.

**Figure 5:**
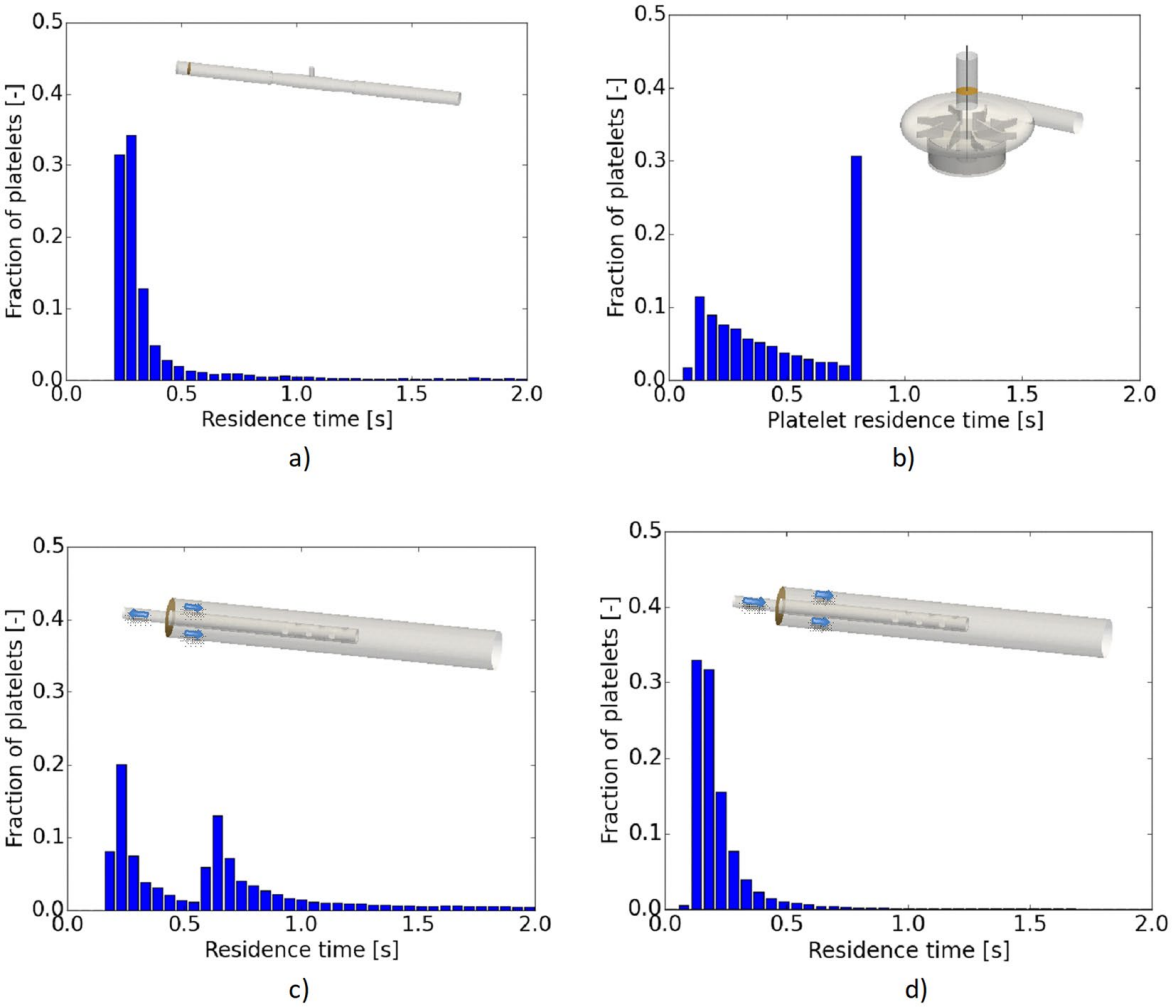
Fraction of platelets vs their residence times for the four cases: (**a**) the connector (**b**) the pump at 3,000 rpm, (**c**) the cannula draining the blood from the patient to the ECMO circuit and (**d**) the corresponding reinfusion case.

As stated above, PAS is dependent on the level of shear-stress and the residence time. The relation between residence time and the activation is depicted in Fig. 5 for the four cases investigated and corresponding to those in Fig. 3. Long residence times were observed for the pump, the drainage cannula and the connector whereas the reinfusion cannula displayed the shortest residence time. Concerning the connector, two groups of platelets were observed (Fig. 5a), with an unexpected increase of number of platelets with longer residence time. This second group is believed to be related to the presence of so called coherent flow structures, i.e. the separation bubble. For the pump, a large increase in number of platelets at a residence time of 0.8 seconds is also noted. This accumulation is due to terminating the tracking of the platelets at 0.8 s, in turn implying that the residence times of almost one third of the platelets will be longer than 0.8 s, i.e. 33 pump revolutions.

**Figure 6:**
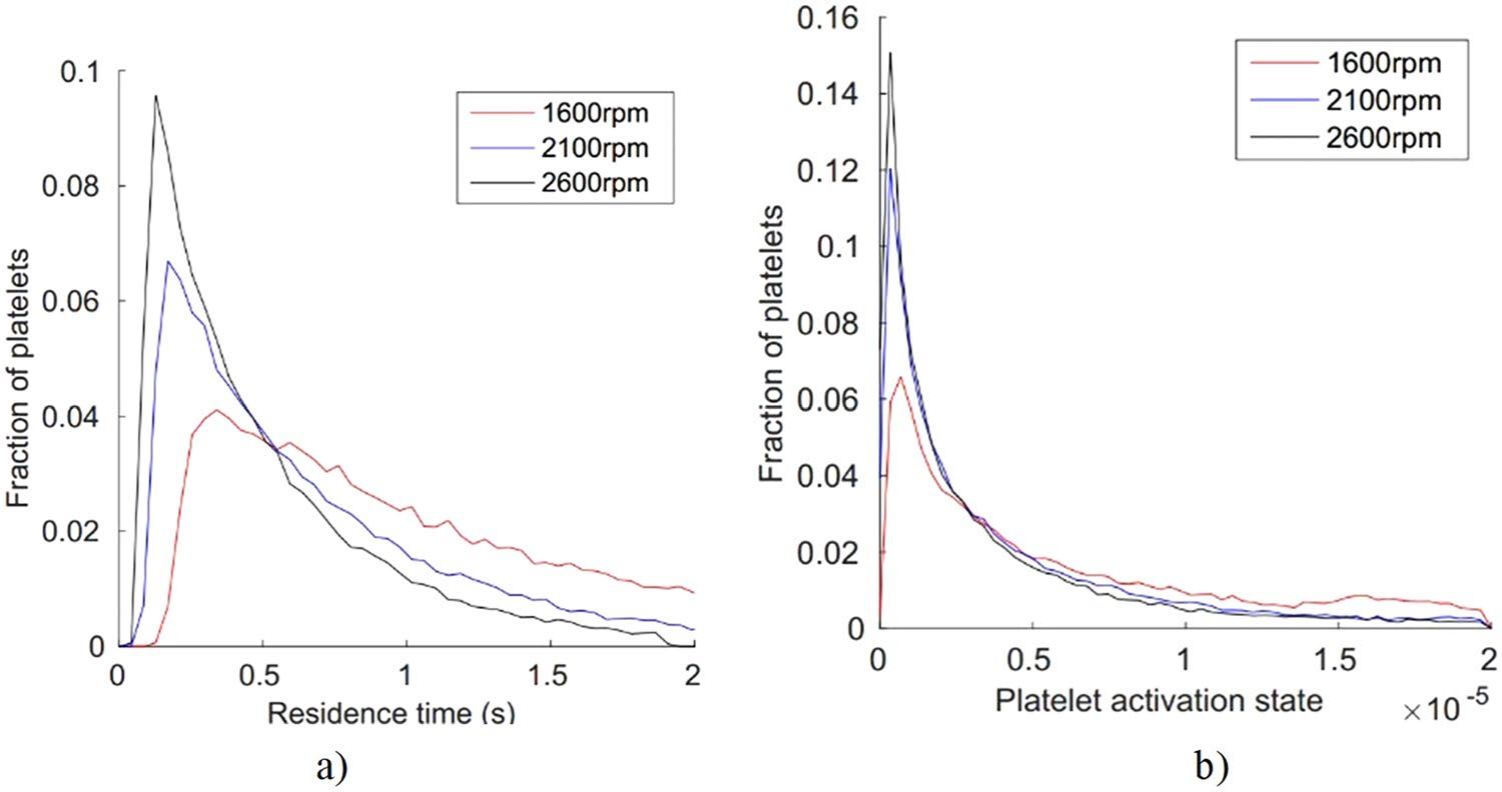
Pump rotation effects as expressed in terms of fraction of platelets vs residence time (**a**) and vs PAS (**b**). Both parameters show larger values at lower rpm on the longer run.

Considering the effect of pump revolutions per time-unit on PAS and residence time. Figure 6 depicts the fraction of platelets vs. platelet residence time and PAS, respectively. This is shown for three different pump *rpm:s*. It is noted that residence time is reduced as the *rpm* increases (Fig. 6a). Increasing *rpm* implies that a larger fraction of the platelets leaves the pump with lower PAS value whereas for low *rpm* the residence time (though with lower shear-stress) will be longer. Hence, more platelets may exhibit higher PAS values (Fig. 6b) during prolonged time exposure at lower *rpm*. This shows that shear-stress levels and residence times are equally important for thrombogenicity of a device. Thus, by increasing pump speed the risks for platelet activation may not necessarily increase.

## Discussion

Predicting formation of thrombus in the ECMO circuit is a great challenge since it should reflect a wide range of biological, chemical and mechanical factors affecting several self-regulating systems with responses and interactions not fully understood. Traditionally, the focus has been on platelet activation, with the assumption that once the platelets are activated the process of thrombus formation is self-generating and irreversible. Here, we address mainly the question of platelet activation, mostly relevant for lower shear-rate situations.

Considering common platelet activation models, the model proposed by the research group led by Bluestein^8–11^ is a feasible approach, whereby the semi-quantitative indicator PAS for the thrombogenicity of a device can be assessed. The model accounts for the accumulated effect of the stress over the path of the platelets. The model is based purely on the accumulative effect of the scalar form of the shear-stress where the three parameters (equation 2) have been determined through empirical calibration. Thus, a global model of the type that we use in this work, does not attempt to model details of thrombus formation process nor the details of the “activation” of the platelet. As the model accumulates the effects of the stress, the outcome does not distinguish between low-stress over longer time or high-stress over shorter time, a shortcoming noted by the group developing the model. Soares *et al*.^9^ introduced the concept of platelet “sensitization”. For flow situations where sub-critical stress acts on the platelet repeatedly, this concept is likely to be important. On the other hand, such model may be more important for describing activation of other cells (e.g. leukocytes and endothelium) having the ability to repair, and with a life-span much longer than that of platelets. The level of repeatability and sensitivity of the results and thereby the sensitivity of the model parameters is not known.

The numerical models used here assume that the carrier fluid is Newtonian (e.g., water). The reason for this assumption was to enable comparisons of the numerical simulations for the three circuit components with experiments (not described here), as the main aim in this study was to determine the relative risks for thrombus formation in the different components. Assumptions concerning the fluid viscosity does not alter the conclusions. Moreover, the same applies in considering the levels of shear-rate and residence times separately as well as using a combined model of the type used herein.

At high shear-rates, the morphology and binding of the vWF to platelets, occurs already at or above 5,000 s^−1^. The observations of Ruggeri *et al*.^13^ suggests that at shear-rates above 10,000 s^−1^, aggregates may develop without platelet activation. As observed in the current study of different ECMO components, higher shear-rates than 10,000 s^−1^ was obtained. This in turn strongly emphasizes the vWF-platelet path of thrombus formation^14^. The results presented indicate the importance of studying not only platelet activation but also emphasizes the importance of the detailed effects of the flow on the vWF. Similarly, it may be expected that at very high shear-rates there is enhanced risk for RBC hemolysis and due to free plasma hemoglobin also induction of thrombus formation^15^.

In spite of its limitations, the model used in the current study may be valuable for evaluating the (relative) risk for coagulation activation by different devices. As indicated by the current investigation, the pump may be the key device for activation of platelets. We identified the regions of high levels of stress and long residence times. Critical locations in the pump are the gaps between the impeller magnet and the pump housing along with the gap between the impeller blades and the roof of the pump house. Due to the narrow gap sizes and the high flow speed difference, cells adjacent to these parts are subject to strong shear-rate. In the magnet housing gap, the observed Taylor-Couette vortices result in longer residence times. The blade-tips also generate strong vortices where the shape of the roof leads to longer residence time. Two other regions of flow separation within the pump are the tongue and the outflow pipe. Also these areas are suspected to be problematic regions. The reinfusion cannula with a lighthouse tip carrying small side holes generate stronger shear as compared to the same design but used for drainage. In combination with low flow rate, the results indicate that the cannula may be a site for thrombus formation. The connector showed the lowest PAS levels although with relatively large shear-rate. The reason for lower PAS levels are associated with the shorter residence time. The flow in the connector was highly fluctuating (turbulent) characterized by a fluctuating shear-layer and a trapped separation bubble. In fact, it is not uncommon to clinically detect thrombi at the downstream part of the tubing connector junction^7^. It is also of note that an ECMO circuit may contain 6–10 connectors. Thus, the accumulated effect of serially arranged connectors in the ECMO circuit may be as significant or more so than that of the cannula or the pump. This finding indicate that one should be restrictive in the use of connectors when constructing the circuit.

In this paper we considered four components of the ECMO circuit. Missing components, such as the oxygenator, heat-exchanger and tubing were considered by Pelosi *et al*.^16^. That paper includes models of the intricate flow in the complex geometry of the oxygenator in addition of using the same type of platelet activation model. It is difficult to assess and directly compare the thrombogenicity of the components studied in this paper and those studied by Pelosi *et al*. However, it would be highly interesting and beneficial to try to quantify the thrombogenicity of all the components of the ECMO circuit.

To adequately study the effects of flow on thrombogenicity, the transport process and concentration distribution of blood cells and macro-molecules functions, high temporal and spatial resolution is essential. Computing only the time-averaged flow characteristics assuming that blood is a homogenous liquid with constant rheological properties is obviously not appropriate for predicting the risk for thrombus formation. The non-uniform concentration of RBC, macro-molecules and platelets are important for the flow characteristics and for thrombus formation. These aspects are to be included in future studies.

## Conclusions

Thrombus formation in the ECMO circuit may occur due to direct platelet activation from shear-stress over time. Thrombi may develop also at high shear-rates, due to the stretching of native vWF multimers whereby activation-independent platelet aggregation may occur, or due to hemolysis. The current investigation showed in a numerical model for platelet activation that volume flow in different ECMO circuit components may impact platelet activation. The largest risk for activation was found for the centrifugal pump head followed by the reinfusion cannula, the drainage cannula and the lowest risk was observed for the straight tubing connector. Long residence time and high shear-rate were the causes for pump thrombogenicity. The connector has high level of shear-rate that may contribute to the formation of aggregates without direct platelet activation but instead through modulation of the vWF at high shear-rate. Both shear-rate and residence time are equally important for platelet activation.

## Electronic supplementary material

Connector LPT

ECMO pump LPT

Drainage cannula LPT

Infusion cannula LPT

ECMO connector velocity

Pump velocity

Supplementary Information
