# Supplementary material for: Flow-induced platelet activation in components of the extracorporeal membrane oxygenation circuit
Source: Sci Rep. 2018 Sep 18;8:13985. doi: 10.1038/s41598-018-32247-y (PMC6143512; doi:10.1038/s41598-018-32247-y)
Supplement: Supplementary file 7 — Supplementary Information [file 41598_2018_32247_MOESM7_ESM.docx]

**SREP-18-16103: Flow-induced platelet activation in components of the extracorporeal membrane oxygenation circuit**

Corresponding author: Gabriel Fuchs

Affiliation: Dept. of Physiology and Pharmacology, Karolinska Institutet,

Stockholm, Sweden.

Postal address: Sundsvalls Hospital, Lasarettsvägen 21, 856 43 Sundsvall Sweden

E-mail address [gabriel.fuchs@ki.se](mailto:gabriel.fuchs@ki.se)

Work telephone number +46730833751

Work fax number +46 8 796 98 50

Co-author: Niclas Berg

Primary affiliation Linné Flow Centre, KTH Mechanics, Royal Institute of

Technology (KTH), Stockholm, Sweden.

Postal address Dept. Mechanics, KTH, 10044 Stockholm, Sweden

E-mail address niber@kth.se

Co-author: L. Mikael Broman

Primary affiliation ECMO Centre Karolinska, Pediatric Perioperative Medicine and

Intensive Care, Karolinska University Hospital, Stockholm, Sweden.

Postal address ECMO Center Karolinska University Hospital Norrbacka, S3:03 S-171 76, Stockholm SWEDEN

E-mail address [lars.broman@sll.se](https://www.mech.kth.se/horde/imp/dynamic.php?page=message&buid=97034&mailbox=SU5CT1g&token=effr67_ppyNn_Z5MYVxvJnN&uniq=1523785322918)

Co-author: Lisa Prahl Wittberg

Primary affiliation Linné Flow Centre, KTH Mechanics, Royal Institute of

Technology (KTH), Stockholm, Sweden.

Postal address Dept. Mechanics, KTH, 10044 Stockholm, Sweden

E-mail address prahl@mech.kth.se

Legend for Supplementary Information

1. **File name: "connectorLPT.avi"**

Platelets motion & activation in a connector:

Note the late platelets with reddish color, indicating activation. These platelets are found mainly downstream of the tube-connector junction (separation bubble and the boundary layer) with long residence times. See Fig 4a in the paper.

1. **File name: "ecmo_pumpLPT.avi”**

Platelets motion & activation in the pump:

The platelets are transported with the fluid, but have longer residence time in the magnet house and near the roof of the pump house. The corresponding platelet paths colored with the level of activation are depicted in Fig 4b in the paper.

1. **File name: "cannula_DrainageLPT.avi”**

Platelets motion & activation in a drainage cannula:

Platelets are injected into the outer vessel. These platelets reach the holes of the cannula and are sucked into it, leading to large PAS values. Some platelets are observed at the end of the animation near the cannula holes. Paths of platelets

with largest PAS are depicted in Fig 4c in the paper.

1. **File name: "cannula_InfusionLPT.avi”**

Platelets motion & activation in a re-infusion cannula Platelets are injected in the cannula and the vessel. Most platelets exit the region shown during the period of the animation. Even platelets with largest PAS are washed out. Paths of platelets with largest PAS are depicted in Fig 4d in the paper.

1. **File name: " ecmo_connector_velocity.avi”**

Instantaneous streamwise velocity field in the connector. Note the strong unsteadiness and the unsteady separated flow region at the downstream tube-connector junction.

1. **File name: " pumpVelocityAnimation_c.avi”**

The animated instantaneous fluid speed field in the pump shows the strong temporal variations of the flow.
